# Enhancing Energy Efficiency and Fast Decision Making for Medical Sensors in Healthcare Systems: An Overview and Novel Proposal

**DOI:** 10.3390/s23167286

**Published:** 2023-08-20

**Authors:** Ziyad Almudayni, Ben Soh, Alice Li

**Affiliations:** 1 Department of Computer Science and Information Technology, School of Computing, Engineering and Mathematical Sciences, La Trobe University, Bundoora, VIC 3086, Australia; z.almudayni@latrobe.edu.au; 2 La Trobe Business School, La Trobe University, Bundoora, VIC 3086, Australia; a.li@latrobe.edu.au

**Keywords:** Internet of health things, edge computing, Fog and Cloud computing, Mist computing, energy efficiency, fuzzy logic, computing capacity, load balancing

## Abstract

In the realm of the Internet of Things (IoT), a network of sensors and actuators collaborates to fulfill specific tasks. As the demand for IoT networks continues to rise, it becomes crucial to ensure the stability of this technology and adapt it for further expansion. Through an analysis of related works, including the feedback-based optimized fuzzy scheduling approach (FOFSA) algorithm, the adaptive task allocation technique (ATAT), and the osmosis load balancing algorithm (OLB), we identify their limitations in achieving optimal energy efficiency and fast decision making. To address these limitations, this research introduces a novel approach to enhance the processing time and energy efficiency of IoT networks. The proposed approach achieves this by efficiently allocating IoT data resources in the Mist layer during the early stages. We apply the approach to our proposed system known as the Mist-based fuzzy healthcare system (MFHS) that demonstrates promising potential to overcome the existing challenges and pave the way for the efficient industrial Internet of healthcare things (IIoHT) of the future.

## 1. Introduction

The Internet of Things (IoT) facilitates seamless communication between sensors and actors within a network, serving specific tasks across various work environments, including healthcare systems. The fundamental objective of IoT networks is to streamline workflows and enhance overall convenience.

The primary objective of IoT networks is to streamline workflows and enhance overall convenience by facilitating real-time data sharing, analysis, and decision making. As a result, the demand for IoT networks is anticipated to undergo a remarkable surge in the upcoming years, with forecasts projecting an astonishing 55.7 billion connected devices by the year 2025 [1]. This exponential growth underscores the importance of ensuring the stability, security, and adaptability of IoT technology to effectively cater to the evolving demands of modern industries. 

Within the healthcare sector, the reliance on IoT network services is poised to become even more pronounced. Such services hold the potential to revolutionize patient care by empowering doctors, nurses, and medical practitioners to closely monitor and manage patients’ health conditions in real time. Through the continuous collection and analysis of patient data from various IoT-enabled devices, healthcare professionals can make informed decisions promptly, leading to quicker responses to critical situations and more effective treatment strategies. Moreover, IoT networks offer the capability to automate administrative tasks, such as scheduling appointments and managing medical records, which not only saves time but also reduces the risk of errors. 

To that end, this paper introduces a pioneering solution known as the Mist-based fuzzy healthcare system (MFHS), which addresses the crucial aspects of processing time and energy efficiency within IoT networks. MFHS leverages the concept of the Mist layer, an intermediate layer that strategically manages the allocation of IoT data resources in the initial stages of data processing. By efficiently distributing computational tasks and data-processing activities, MFHS optimizes the utilization of resources, leading to reduced processing times and enhanced energy efficiency. This innovative approach has the potential to significantly improve the overall performance of IoT networks, ensuring that healthcare systems and other industries can leverage the full benefits of IoT technology. 

The paper comprises six sections, where the first section critically examines and analyses recent studies relevant to MFHS. The second section provides a comprehensive review of previous studies that leveraged fuzzy logic systems to address IoT network challenges. Section 3 offers an overview of the motivation and contributions of MFHS, while Section 4 presents the detailed methodology employed in MFHS. Section 5 showcases the results obtained from the implementation of MFHS, including a comparative analysis with existing approaches. Finally, the concluding section summarizes the key findings and highlights the implications of MFHS in advancing IoT network efficiency within healthcare systems. 

## 2. Related Work

We undertake a thorough examination of the relevant literature, which forms the foundational segment of our research. Through a discerning analysis of existing works, we identify pertinent shortcomings and gaps, setting the stage for the subsequent phase of our study. Building upon the insights gleaned from our critical review, the subsequent segment of our research unfolds as we introduce an innovative solution in Section 3 and Section 4. Drawing from the identified issues, we present a novel framework that addresses these concerns and offers a promising avenue for advancement.

The related work itself consists of two parts:

(A) The load balancing of IoT networks among the four layers (Edge, Mist, Fog, and Cloud).
(B) The effective use of fuzzy logic systems in networking.

### 2.1. Load Balancing among the Four IoT Network Layers

This section provides an overview of recent studies conducted between 2019 and 2022 that focused on workload balancing and task management within IoT systems across the Edge, Mist, Fog, and Cloud layers. The objective of these studies was to leverage the capabilities of all four layers and optimize their utilization within the system. However, this section also highlights the existing gaps in these studies and proposes potential solutions to address them.

In their work, Barik et al. (2021) [2] introduced a new algorithm known as the RAO-1 ALGORITHM, which aimed to minimize energy consumption within the Mist computing environment of IoT networks. The proposed approach consisted of two stages: reducing the number of unutilized microcomputers and effectively allocating tasks to suitable participating microcomputers. By implementing the algorithm using Python 3.7, the results demonstrated reduced power consumption and superior performance compared to the ECTC and MaxMaxUtil algorithms. However, from our perspective, simply reducing the number of unused microcomputers within the Mist layer may not provide an effective solution. It would be more beneficial for the authors to focus on effectively utilizing all available microcomputers and achieving workload balance to alleviate the burden on the Cloud and Fog computing environments. Furthermore, considering factors beyond just task completion time, such as data size, can contribute to making better resource allocation decisions.

In traditional IoT networks using the MQTT communication protocol, the MQTT brokers are distributed in the network and concentrated to Cloud nodes. Thus, the traditional MQTT protocol is inappropriate for time-sensitive applications because it does not support real-time communication between IoT devices. Therefore, Hmissi et al., 2021 [3] proposed a new approach called MQTT-MBD to meet task deadlines and improve network efficiency by distributing MQTT brokers over Mist nodes. To ensure that MQTT-MBD improves communication delay, the authors identify four steps: registration, selection, assignment, and connection, to select an appropriate broker. The broker selection is based on three criteria: enough energy, meeting the real-time constraint, and CPU utilization. Finally, a task will be sent to the Mist broker only if the Cloud broker, the Fog broker, and Edge broker cannot answer the deadline. The simulation results showed that MQTT-MBD achieved better results than Extended-EMMA and DM-MQTT in energy efficiency and delay. The only notable drawback that can be seen in this proposed approach is that the process of selecting an MQTT Mist broker to control tasks for the systems comes at a very late stage. This is because IoT tasks must travel to all layers in a decreasing order, starting from the Cloud to the Mist layer, and ask each computing layer if it can meet the deadline requirements for the task until it reaches the Mist layer. This process increases the execution time and consumes power; however, these circumstances can be avoided if selecting the Mist broker comes earlier.

Resource allocation and managing tasks among Cloud, Fog, and Mist computing in the IoT networks is a challenge. Therefore, Refaat 2020 [4] proposed a new model called multi-level IoT tasks scheduling (MLITS) to manage and allocate resources for IoT tasks and distribute these tasks over the Cloud, Fog, and Mist computing environments. The criteria to distribute tasks among these computing environments is based on the task’s deadline and the urgency to execute it. Using CloudSim 3.0.2, the proposed approach succeeded in achieving better results than the Min-Min, CBS, and EFDF algorithms in terms of the time to complete a task, as well as having less waiting time and more significant throughput. However, the MLITS has some drawbacks; the criteria to distribute tasks is limited, especially when it is known that the Fog, Cloud, and Mist have different source capabilities. The second drawback is that all IoT tasks are first sent to the Mist, then if the Mist nodes are not capable of processing a task, it will be offloaded to the Fog nodes, and so on, until it reaches the Cloud. It is more optimal for a task to directly find its resources, as the offloading process incurs energy costs.

Due to the limited resources of Mist computing nodes, they are incapable of handling all IoT tasks, necessitating the offloading of these tasks to the Fog or Cloud for further processing. Hence, it is crucial to be aware of the node capabilities and the task requirements in all layers. Thus, Drosdov et al., 2019 [5] proposed a new model to extend the offloading process to Mist computing. The authors implemented two services to offload tasks from one to another accurately. First, the node summarizer (NS) collects information about the hardware capabilities. Second, the service summarizer (SS) gathers information about the service requirements. These two services help in selecting the optimum Mist node when offloading tasks. The proposed model was applied in three different experiments; the results showed that it successfully added 20 ms processing time to the system. In this proposed approach, the authors only focused on offloading tasks from one Mist node to another Mist node in the system and ignored all other layers. Furthermore, the task offloading criteria solely focus on analysing the Mist nodes, without taking into consideration any specific concerns related to the tasks themselves.

Hensh et al., 2021 [6] studied and analysed the impact of extending the computing process through the Cloud, Edge, and Mist layers to balance the workload among them and engage all layers to be a part of the computing process. The authors divided the priority of tasks into four types to select a resource. The classification is based on the time needed to complete a task and the response time’s importance level. Using Visual Studio code, the authors completed four different experiments to compute tasks: Edge only, Cloud only, Fixed Edge–Cloud, collaborative Edge–Cloud and Mist–Edge–Cloud. The simulation results proved that the experiment of Mist–Edge–Cloud achieved better delay time than the other experiments. The authors did not introduce a new algorithm; instead, they conducted an experiment to highlight the significance of utilizing all four computing layers in data computation.

Shahid et al., 2021 [7] implemented a new framework called IoTNetWar for military organizations to achieve better time and resource scenarios when using IoT service monitor troops. The authors take advantage of machine learning; specifically, they employed the delay-based K-nearest neighbour algorithm to distribute IoT tasks among the Mist, Fog, Cloud, and application layers. By employing this ML algorithm, a task embarks on a journey from Mist computing to the Cloud, seeking an appropriate resource. EdgeCloudSim was used to validate the proposed framework on three different scenarios: Fog computing (Load-balanced), Mist computing (Load-balanced) and Mist computing delay-based K-nearest neighbours (KNN). The results proved that the proposed framework of Mist computing (KNN) achieved better results than Fog computing (LB) and Mist computing (LB) in terms of service time, processing time, latency, and CPU utilization. It is noticeable from the framework that there is no resource allocation in the IoTNetWar algorithm, as tasks search for a suitable resource to fit task requirements starting from the first layer (Mist layer) to the last layer (Cloud layer). The process of searching for resources for IoT tasks among all layers is very time-consuming, and this can be avoided if the resources are selected at an early stage without the need for searching.

Resource management and allocating resources for IoT tasks accurately is one of the keys to achieving better latency, bandwidth, and energy efficiency for IoT networks. Therefore, Hosen et al., 2022 [8] proposed a new algorithm called MSRM-IoT to allocate resources for IoT tasks. All IoT tasks are first sent to the Edge broker (EB) in this algorithm. Inside this EB, there is an application receiver and classifier (AC), and its function is to distribute these tasks among Mist computing, Fog computing, and Cloud computing based on the input size, the number of tasks, and the workload of a task in MIPS. In addition to the AC, there is a Mist resource manager in the EB to assist tasks to select the best Mist node. Moreover, there is a Fog broker (FB) in the Fog computing layer, and its function is similar to EB. MATLAB19a was used to validate the proposed algorithm and compare it with three evolutionary algorithms: router, FCFS, and short job first. The results showed that MSRM-IoT outperforms the three algorithms across all measures [8]. The parameters mentioned in MSRM-IoT are mainly based on the computing size only to determine a resource for the IoT tasks, which is not efficient from our point of view. In allocating resources for IoT tasks, it is essential to determine tasks’ importance and their timing requirements in real-time processing.

Ejaz et al., 2020 [9] analysed the difference between computing IoT tasks in the traditional Cloud-IoT model, Edge-Cloud-IoT model, and local Edge-Cloud-IoT model. iFogSim was used to evaluate the three different scenarios. The simulation results showed that processing IoT tasks locally is the best with respect to connectivity and energy consumption compared with the first and second models. However, it is important to know that different IoT tasks might vary in different scenarios, and processing tasks locally is not always practical, as local nodes have limited capabilities. Thus, Cloud and Fog assistance is always required to serve in all scenarios.

Tripathy et al. (2022) [10] introduced the Secure-M2FBalancer model to enhance the security and workload balancing of IoT networks through the integration of supervised learning and a genetic algorithm. The proposed model was implemented within a healthcare management system and comprised four layers: the IoT layer, Mist layer, Fog layer, and Cloud layer. In this model, the IoT layer forwards data to the Mist layer, which is responsible for resource allocation. If the Mist layer cannot process the data, it is offloaded to the Fog layer. Within the Fog layer, a technique is employed to determine the status of servers, distinguishing between overloaded and underloaded servers to enable accurate resource allocation. Furthermore, encrypted data are offloaded to or received from the Cloud layer for secure communication when additional processing or storage is required. The proposed approach was validated using MATLAB, and the results demonstrated superior makespan performance when compared to least association, round-robin, and weighted round-robin approaches. However, within the Secure-M2FBalancer model, the authors solely considered response time as the primary factor for resource allocation at the Mist layer. From our perspective, this single factor may not be sufficient for precise decision making in server allocation. Therefore, we suggest the inclusion of additional factors to enhance the accuracy of the decision-making process. Furthermore, the criteria used to determine the state of Fog servers, whether overloaded or underloaded, are not explicitly defined in the study.

Mist computing can play a vital role in improving the data collection for IoT devices as the processing is in the Edge computing environment. Barik et al., 2017 [11] developed a new platform called MistGiS for geospatial big data and applied it in two cases: tourism information infrastructure management and a faculty information retrial system. The Raspberry Pi microprocessor was utilized to build the framework. The study results found that Mist computing can assist Cloud and Fog computing in providing better analysis for geospatial big data. This platform serves as a means to only highlight the significance of Mist computing in evaluating other layers, thus lacking novelty in its approach.

Stavrinides et al. (2021) introduced a scheduling heuristic that considers security and cost for real-time IoT data processing, taking into account various security requirements. The algorithm is divided into three stages:

1. In the task selection stage, tasks are prioritized based on their deadlines.
2. The VM filtering stage aims to select the appropriate security level.
3. The VM selection stage determines the suitable VM for task processing based on the earliest estimated finish time.

To validate their approach, the researchers implemented a custom discrete-event simulator in C++. The results of the study demonstrated that the proposed approach outperformed the baseline policy of security-aware heuristics (SAH) in terms of deadline miss ratio, total cost of Cloud resources, and average response time [12]. However, we believe that additional factors, such as task size, should be considered when prioritizing tasks. 

### 2.2. Fuzzy Logic and Load Balancing

In this section, we provide an overview of previous studies conducted between 2018 and 2021 that leveraged the fuzzy logic system to enhance decision making in their respective approaches. These studies have been compiled to showcase the efficacy of employing fuzzy logic in making accurate decisions pertaining to task scheduling and load balancing in IoT systems.

Ali H et al., 2021 [13] proposed a fuzzy logic algorithm called real-time task scheduling (FLRTS) to enhance the execution of the tasks of IoT applications at the Fog layer. The algorithm works as a filter to divide the tasks into two categories: Fog group and Cloud group, to select the environment to execute data to either the Fog or the Cloud. Furthermore, the algorithm works inside the Fog broker at the Fog layer for the purpose of classification. The algorithm incorporates five inputs: CPU utilization, storage utilization, bandwidth utilization, task deadline, and network latency, to determine the optimal execution environment for data, whether it should be processed in the Fog or the Cloud. To evaluate the proposed algorithm, the IFogSim simulator, a Java-based simulation toolkit, was employed. The simulation results showed that the proposed approach outperforms the existing algorithms first in first out (FIFO) and short job first (SJF) scheduling algorithms and real-time task processing (RTP) in terms of makespan, delay, success ratio, and average turnaround time. To achieve improved response time and decision making, it is crucial for the filtering process to be situated closer to IoT sensors rather than at the Fog layer. Filtering should ideally take place at an early stage, and in our perspective, the Mist layer is the most suitable for task filtering since it is the layer closest to the Edge layer (which represents the sensors layer).

Das A et al., 2020 [14] proposed a user categorization using a fuzzy logic (UCFL) algorithm to improve the overall performance of IoT networks. The framework consists of three layers: the second layer (Fog devices) works as a chine to allow the first layer (users) to collect data from the third layer (sensor nodes). Due to the power constraints in the IoT network, the sensor nodes are not involved in the authentication process to save power. Instead, only nodes with higher capacity, specifically the Fog nodes in this framework, are engaged in the authentication process. The algorithm takes into account three inputs: user experience, user knowledge, and user recommendation, to generate an output that classifies users into three categories: high-trusted, medium-trusted, and low-trusted. This classification plays a crucial role in reducing the number of authentication phases required. High-trusted users do not require authentication phases as they have high experience, knowledge and recommendation, while medium-trusted users only require one phase of authentication. On the other hand, low-trusted users require two phases of authentication as they have low experience, knowledge, and recommendation. To evaluate the proposed algorithm, a Raspberry Pi was employed as a user, while a laptop served as a Fog node to facilitate the communication process. The simulation results clearly demonstrated that the proposed approach outperformed other algorithms such as CoAPMicro, CoAPBlip, and HTTP/UDP in key metrics including handshake duration, memory consumption, average response time, and computation cost. In the UCFL framework, the authors achieved performance improvements by reducing certain users’ authentication processes and adjusting security levels. However, it is important to note that from our perspective, enhancing network performance should not compromise the system’s security, either directly or indirectly.

In their work, Reddy A et al. (2021) [15] proposed an algorithm called the feedback-based optimized fuzzy scheduling approach (FOFSA) to enhance power efficiency, execution time, and makespan in IoT networks while meeting quality of service (QoS) requirements. The algorithm intelligently allocates appropriate resources from Cloud resources, including virtual machines, applications, and data centres, to compute tasks. Resource estimation for Cloud computation takes place at the Fog layer. The proposed algorithm was evaluated using MATLAB 2017b, with a focus on applying it to a rainfall prediction mechanism. The algorithm takes five inputs: clouds, tasks, expected completion time, user set priority, and decision making, and generates rainfall predictions by estimating suitable VMs. Simulation results demonstrated that the FOFSA approach effectively reduced power consumption, execution time, and makespan compared to existing algorithms such as the optimized fuzzy bee-based scheduling algorithm (OFBSA); dynamic duty scheduling for green sensor Cloud applications (DDSA), an adaptive tasks distribution method for green Cloud computing; and the osmosis load balancing algorithm (OLB). However, one limitation of the FOFSA algorithm is the lack of criteria and measurements to determine input values. For example, when considering the time to complete a task, there is no standard measurement to classify it as low or high, which may impact the accuracy of the algorithm. Additionally, the computing capacity of resources was not explicitly considered in the algorithm.

Cuka M et al. [16] 2018 proposed two fuzzy logic algorithms to select IoT devices in opportunistic networks for task computing. The challenges that opportunistic networks face are considered to determine the inputs for the two fuzzy logic algorithms, namely FBS1 and FBS2. In FBS1, there are three inputs: the waiting time, the storage, and the remaining energy to assist in generating an output for the IoT device to process in an opportunistic network. In FBS2, one new parameter is added to the three inputs in the FBS1 to achieve the same objective. The FBS1 is less complex than the FBS2; however, the simulation results demonstrated that the FSB2 achieved better results than the FBS1 in selecting IoT devices. One drawback of this approach is that the authors primarily focused on selecting a suitable source for data computation without considering the characteristics of the data itself.

In their work, Haripriy A et al. (2019) [17] introduced a lightweight fuzzy logic algorithm called Secure-MQTT to enhance the security of IoT applications, specifically in healthcare monitoring, by utilizing the MQTT communication protocol. The algorithm incorporates an efficient intrusion detection system to safeguard IoT applications against denial of service (DoS) attacks. The Secure-MQTT algorithm takes two inputs, namely the connection message ratio (CMR) and connection acknowledgment message ratio (CAMR), to detect anomalous behaviours and generate an output indicating whether the behaviour is normal, abnormal, or indicative of an attack. To evaluate the proposed approach, the Contiki simulator COOJA was employed. The simulation results demonstrated that Secure-MQTT exhibited superior performance in detecting attacks compared to existing methods, thereby enhancing the overall security of IoT applications.

In their study, Khalil A et al. (2019) [18] presented a fuzzy logic algorithm designed to assess the trust level of nodes for data collection purposes. The algorithm’s evaluation of node trust is based on three inputs: device physical security, device security level, and device ownership trust. These inputs are processed by the fuzzy logic system to generate an output, which represents the trust rating of a node on a scale from one to ten. A rating of one indicates the lowest level of security, while a rating of ten indicates the highest level of security. To assess the proposed approach, the researchers utilized FISpro 3.5 and conducted evaluations on various scenarios involving different input values. The simulation results revealed that IoT nodes experienced increased trust levels when the inputs had higher parameter values, signifying better security attributes for the selected nodes.

Sankar S et al., 2018 [19] proposed a fuzzy logic-based energy-aware routing protocol (FLEARPL) to prolong the network lifetime and reduce the power consumption of IoT applications while using RPL. The algorithm assists in selecting the best route during transmission via RPL. It uses three inputs: the routing metrics load, residual energy (RER), and expected transmission count (ETX), to estimate the quality of routes for better selection. The Cooja simulator was used to evaluate the proposed algorithm. In comparison with RPL, MRHOF-RPL, and FL-RPL, the simulation results showed that the proposed algorithm outperforms them in terms of the packet delivery ratio and network lifetime.

Ramkumar K [20] proposed fuzzy-based relay node selection and energy-efficient routing (FRNSEER) to enhance QoS performance in WSNs. The FRNSEER selects the best sink node among active relay nodes to collect data from sensor nodes; sensor hubs were applied to link sensor nodes to the sink. Energy and utility were the parameters to determine active nodes. After defining the active nodes, fuzzy logic rules were applied to select the best node from the active nodes to work as a sink node. The selection of the sink node was based on three inputs: virtual distance (VD), virtual energy (VE), and node bandwidth. A network simulator (NS2) was employed to evaluate the proposed approach. The simulation results demonstrated that the proposed approach achieved better results than the fuzzy-based hyper round policy (FHRP) and neural network-based localization scheme (NNBLS) in terms of energy efficiency and packet rate.

In their study, Radhika S et al. (2021) [21] introduced the fuzzy-based sleep scheduling algorithm, which incorporates machine learning techniques to enhance the energy efficiency of wide area networks (WANs). The primary objective of the algorithm is to minimize the message transmission overhead, thereby reducing energy consumption and extending the overall network lifetime. This is achieved through a simplified cluster restructuring process. The algorithm employs machine learning techniques to reduce data transmission, along with two fuzzy logic algorithms that estimate cluster updates and sleep cycles. Inputs such as distance, residential energy, and data rate are utilized in the fuzzy logic algorithms to make accurate estimations. When the node detects similar data, it switches to sleep mode to conserve energy. The proposed approach was evaluated using MATLAB. Simulation results demonstrated that the proposed approach surpasses existing methods such as data density correlation degree (DDCD) and energy-efficient clustering with correlation and random update (EECRU) in terms of network lifespan. The fuzzy-based sleep scheduling algorithm, with its integration of machine learning and fuzzy logic techniques, offers significant improvements in energy performance and network longevity.

## 3. Motivation and Aims: Proposed Model

Previous research in IoT systems, specifically in the areas of fog computing and cloud computing, has focused on task distribution and load balancing among computing layers. The primary goal has been to reduce power consumption and processing time by offloading tasks from one layer to another. However, the offloading process itself requires power, time, storage, and computing capacity. Therefore, it is crucial to minimize the offloading process in order to save energy, time, storage, and computing resources in the IoT system. However, this reduction must be achieved without negatively impacting the system, such as causing delays.

The proposed approach aims to reduce the offloading process by distributing tasks among computing layers in the early stages and leveraging this reduction. By prioritizing tasks and allocating resources to them at an early stage, we can achieve our objective, as tasks will be sent directly to their designated resources unless exceptional cases arise, such as when the resources are full. To achieve this, we allocate resources for tasks processed at the Mist layer. The Mist layer, located close to the Edge layer (medical sensors), plays a crucial role in assisting patients with critical conditions and facilitating real-time monitoring for quick decision making.

When allocating resources, we consider two main factors that help us make better decisions and minimize task offloading. The first factor relates to the health condition of the patients, while the second factor pertains to the capacity of the resources. In this context, we only consider the Mist nodes and the Fog nodes, and we do not take into account the capacity of the Cloud since it is centralized. By leveraging fuzzy logic systems, which are powerful tools for decision making and delivering accurate results, we can estimate and predict the optimal resource allocation for healthcare tasks. To study and analyse the proposed system, we will adopt healthcare systems as a model, focusing on the MFHS (Mist-based fuzzy healthcare system).

### 3.1. Our Proposed MFHS Aims to Achieve the Following

- Decision making at the extreme edge of the network, facilitated by the Mist broker, to enable fast decision making and reduce processing time.
- Estimating patients’ healthcare conditions and allocating resources based on their conditions.
- Prioritizing data packets for patients with critical conditions, ensuring they are served first.
- Minimizing transfer time by allocating resources at the Mist broker, located at the extreme edge of the network.
- Reducing power consumption by eliminating the need for data offloading at all layers except the Mist layer.

### 3.2. The Proposed Approach

**Figure 1:**
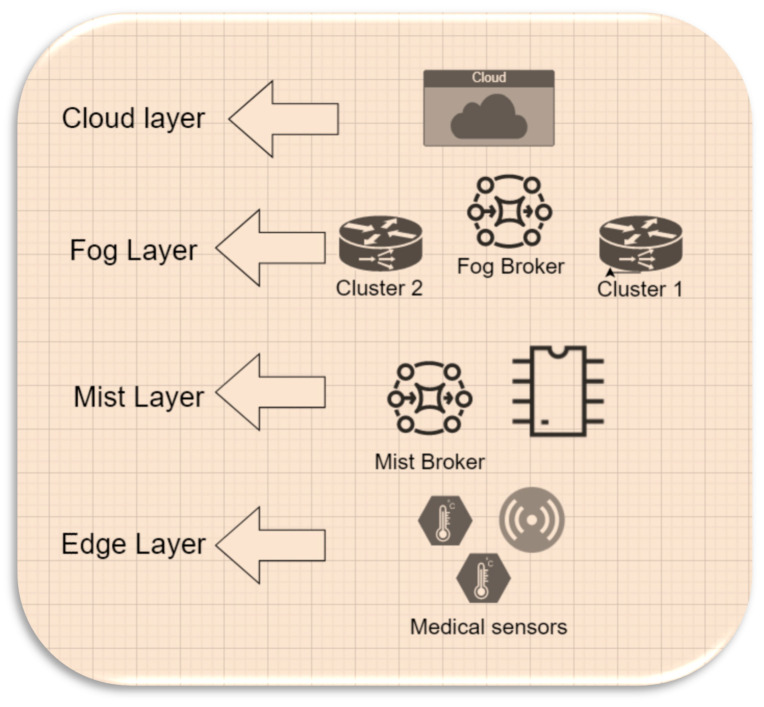
Proposal design.

The MFHS (Mist-based fuzzy healthcare system) operates across four layers: the Edge layer, the Mist layer, the Fog layer, and the Cloud layer, each playing a crucial role in processing data for healthcare systems. In the following section, we provide a detailed description of these layers and outline their functionalities within our approach. Additionally, Figure 1 provides a concise overview of the system design, illustrating all the components utilized in our proposed approach.

(1) Edge layer: It collects medical data such as body temperature using sensor devices. The Edge layer sends the sensed data to the Mist layer, which categorizes data based on the patient’s condition. The Edge layer only sends the sensed data that a Mist layer requires for categorization, which means some medical sensor devices can be removed without affecting the system.
(2) Mist layer: The Mist layer receives the sensed data from the Edge layer. It categorizes data based on the patient’s health condition and the computing capacity of Mist using two fuzzy logic systems, namely MFHS1 and MFHS2. MFHS1 focuses on data categorization, where the Mist broker employs fuzzy rules to classify the data based on the patient’s health condition and its priority. On the other hand, MFHS2 is responsible for estimating the computing capacity of the Mist nodes, enabling the system to determine whether the data should be processed in the Fog, Cloud, or within the Mist layer itself.
(3) Fog layer: The Fog layer via the Fog broker is responsible for exceptional cases, such as when the Mist layer is unable to process data due to storage or capacity limitations. The Fog broker takes charge of distributing these data among the Fog nodes based on the clustering of these nodes.
(4) Cloud layer: The Cloud layer receives high-priority cases directly from the Mist layer for processing. Additionally, it acts as a recipient of data when the computing capacity of both the Mist and Fog nodes is insufficient to handle the workload.

## 4. Phases of the Proposed Approach

**Figure 2:**
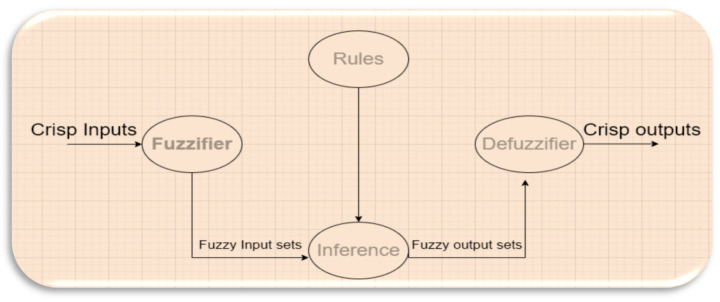
Fuzzy logic system structure.

Before we go further into the design, it is essential to define the fuzzy logic system. Fuzzy logic is a predicting system to make accurate decisions based on fuzzy rules. Figure 2 shows how a fuzzy system works. The fuzzy logic system consists of two main phases: fuzzification and defuzzification. 

Fuzzification converts crisp values to fuzzy set values; in this stage, it is essential to draw the membership function and fuzzy sets and use the established fuzzy rules to generate fuzzy set outputs [22]. Many types of membership functions convert crisp data to fuzzy sets; however, in this design, the triangular membership function is selected as it provides accurate results and suits the design.

The triangular function is given below:

(1) $$\mu_{A}\left(X\right)=\left\{\begin{array}{c} 0\;\;\;\;\;\;\;\;\;\;\;\;\;\;\;\;\;\;\;\;\;\;\;\;x\leq a\\ \frac{x-a}{m-a}\;\;\;\;\;\;\;\;\;\;\;\;\;\;\;\;\;a<x\leq m\\ \frac{b-x}{b-m}\;\;\;\;\;\;\;\;\;\;\;\;\;\;\;\;\;m<x<b\\ 0\;\;\;\;\;\;\;\;\;\;\;\;\;\;\;\;\;\;\;\;\;\;\;\;\;\;\;x\geq b \end{array}\right.$$

where *X* is the new input that requires decision making; and *a*, *m*, and *b* are the points to determine the interval of each triangle in the membership function, with $\mu_{A}\left(X\right)$ as the output of Equation (1).

Defuzzification converts the output values of the fuzzy set into crisp values by using some linguistic rules of if-then and logical operators [23]; in this design, we will use the AND operator. There are many equations to calculate defuzzification, and the centroid of area (CoA) is used in this design. The defuzzification equation is as follows:

(2) $$x^{*}=\frac{\sum_{i=1}^{n}x_{i}\times \mu (x_{i})}{\sum_{i=1}^{n}\mu (x_{i})}$$

Our proposed approach consists of two phases. Phase 1 assists in data categorization. Phase 2 assists in allocating resources depending on the categorized data in phase 1 and Mist node capacity.

### 4.1. Phase 1

The first phase of our proposed system occurs within the Mist broker, where data categorization takes place using a fuzzy system (FS). This process aims to effectively handle patient data across various health conditions and determine the appropriate server resource from the Mist, Fog, and Cloud layers. The Edge layer focuses on three medical sensors, namely the body temperature (BT) sensor, glucose level (GL) sensor, and heart rate (HR) sensor, which record the patients’ health data. These recorded data are initially transmitted to the Mist layer, specifically the Mist broker, where they undergo categorization using the FS. The Mist broker classifies the patients’ health data into three priority levels: high priority (critical cases), medium priority (susceptible to disease), and low priority (healthy), based on the patients’ health conditions.

The FS can accomplish that by converting the actual data (crisp inputs) into linguistic values (fuzzy input set) using the fuzzification method to determine and estimate patients’ health conditions based on fuzzy rules to generate fuzzy output sets. Then these outputs are defuzzified to convert linguistic values to crisp values and count them as the results of the FS. The results of this defuzzification could be of one of the three priorities: high priority, medium priority, and low priority. Table 1 represents how we estimate the health condition of patients.

**Figure 3:**
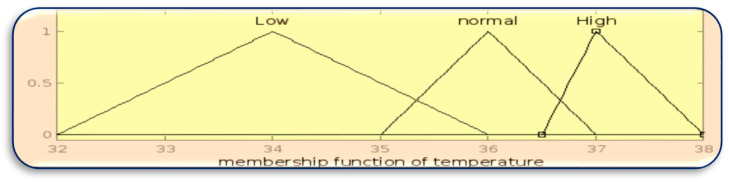
Membership function of BT.

**Figure 4:**
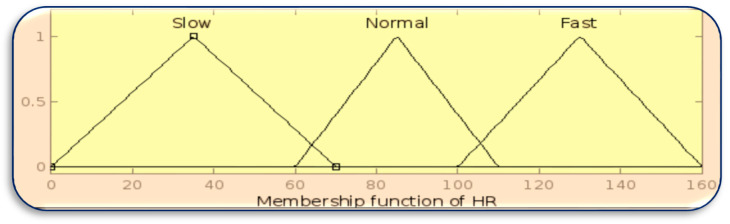
Membership function of HR.

**Figure 5:**
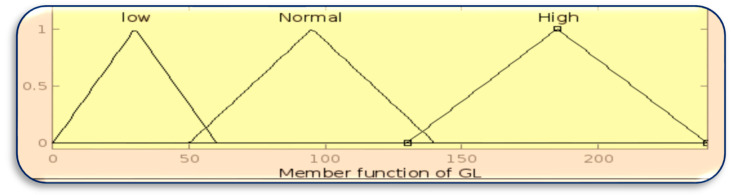
Membership function of GL.

The membership function for inputs BT, HR, and GL is designed using the MATLAB Fuzzy Toolbox in Figure 3, Figure 4 and Figure 5, respectively.

In Phase 1, the number of rules in the fuzzy logic system depends on the number of sensors in the experiment and how many readings each sensor can sense, as follows.

The number of rules = (number of readings BT) × (Number of readings HR) × (number of readings GL).

**Figure 6:**
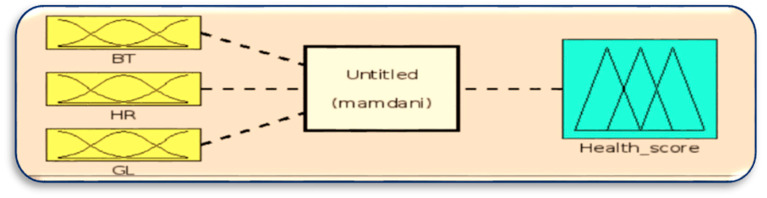
FLS for health score.

**Figure 7:**
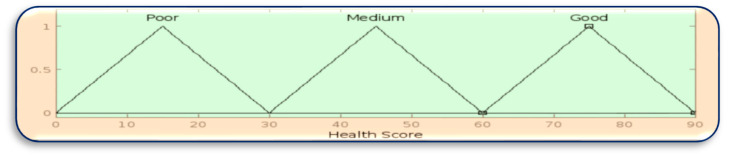
Membership function of health score.

Here, the number of rules = 3 × 3 × 3 = 27 rules by using “if-then” and “and” linguistic rules as logical operators to take the minimum membership value. The rules are represented in Table 2. In the health score calculation, each normal health condition is counted as 30 points, with medium 10 points and low 5 points. Figure 6 shows the design of the fuzzy logic system to generate the health score to assist in data categorization, and Figure 7 shows the membership function of the health score.

### 4.2. Phase 2

In the second phase, the Mist broker (MB) focuses on the Mist’s computational capacity (see Equation (3) below) and data priority to allocate resources for healthcare services. MB selects one of the three resources: Mist, Fog, and Cloud, to provide services for the healthcare system as follows. 

First, MB directly transfers high-priority data to the Cloud and allows healthcare providers to access these critical data. In addition, MB helps patients with urgent conditions in real-time processing to make quick decisions as the MB is very close to the sensing devices. 

Second, MB sends the medium-priority data to the Mist nodes of available capacity. The data are transferred to the next available Mist node if a Mist node is overloaded. If all Mist nodes have insufficient computing space for the medium-priority data, the data are transferred to the Fog broker. 

Third, MB sends the low-priority data to the Mist nodes of available capacity. The data are transferred to the next available Mist node if a Mist node is overloaded. However, if all Mist nodes lack adequate computing space (i.e., low and medium computing capacity) for low-priority data, they are then directed to the Fog broker for further processing, as indicated in Table 3. 

Moving to the subsequent layer, the Fog broker is equipped with a load balancer responsible for distributing the medium- and low-priority data received from the Mist broker among the available Fog nodes. This distribution is based on two factors: the remaining computing capacity of each Fog node and a clustering technique. The calculation of the remaining capacity of a Mist node, as described by Equation (3), plays a crucial role in determining whether the healthcare services should be computed within the Mist node itself or whether the healthcare data should be redirected to another Mist node or the Fog broker in the event of an overloaded Mist node:

(3) $$\mathrm{Remaining}\;\mathrm{capacity}=C-\sum_{i=1}^{n}\mathrm{Pi}\times Si\;\mathrm{Remaining}\;\mathrm{capacity}\;\mathrm{percentage}=\frac{C-\sum_{i=1}^{n}Pi\times Si}{C}\times 100$$

The equation consists of four factors to determine the computing capacity of a Mist node:

- C is the capacity of a Mist node.
- Pi is the packet arrival rate for i as a data packet.
- Si is the size of the data packet i.
- n is the number of data packets.

In Phase 2, the number of rules in the fuzzy logic system depends on the number of sensors in the experiment and how many readings each sensor can sense as follows:

The number of rules = (number of data priority levels) × (number of computational  capacities of Mist node)

Here, the number of rules = 3 × 3 = 9 rules by using linguistic rules of “if-then” and “and” as logical operators to take the minimum membership value. The rules are represented in Table 3.

**Figure 8:**
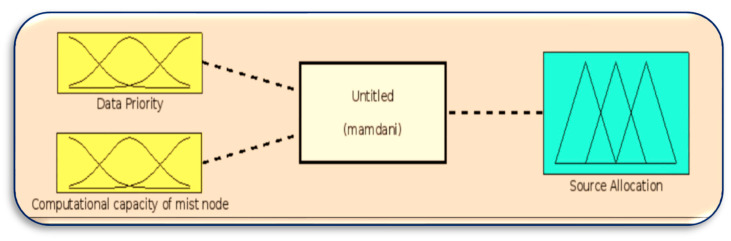
Fuzzy logic system for server allocation.

**Figure 9:**
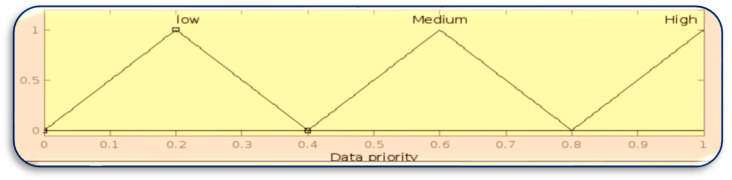
Membership function for data priority.

**Figure 10:**
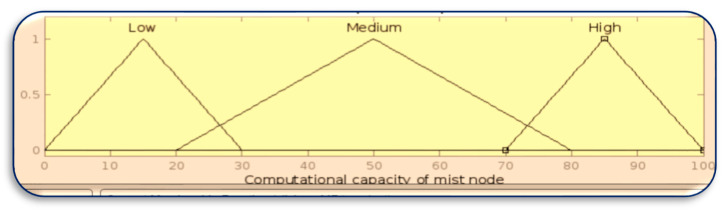
Membership function for Mist capacity.

**Figure 11:**
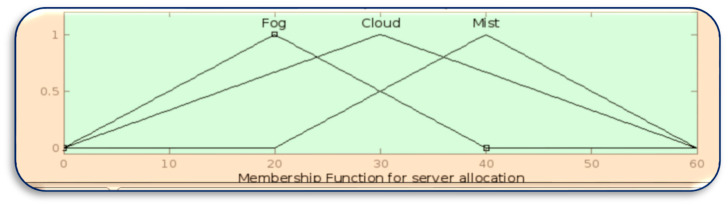
Membership function for server allocation.

Figure 8, Figure 9, Figure 10 and Figure 11 in MATLAB depict the design of the fuzzy logic system responsible for server allocation. This system takes two inputs, namely data priority and Mist capacity, and generates an output that determines the server allocation. 

### 4.3. Fog Broker

**Figure 12:**
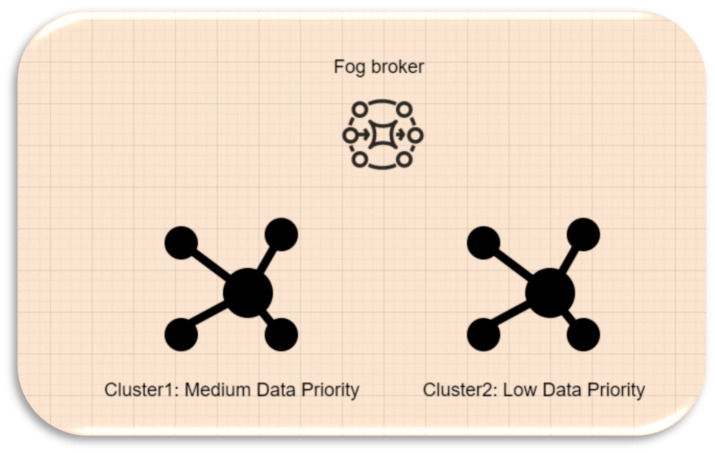
Fog node clusters.

The Fog broker receives only two types of data: low-priority and medium-priority, which are passed on by the Mist broker. To address this distinction in data types, we have designed a clustering scheme within the Fog layer. The Fog nodes are divided into two clusters, with the Fog broker overseeing their operation. Figure 12 provides an overview of the workflow within the Fog layer. Cluster1 is responsible for computing the medium-priority data, while Cluster2 handles the low-priority data. This categorization aims to minimize execution time and the offloading process by assigning appropriate resources to each data type based on priority. To achieve this, the Fog nodes are divided into clusters based on their remaining computing capacity. The first cluster comprises the Fog nodes with higher remaining capacity, while the second cluster consists of nodes with lower remaining capacity. The decision-making process for medium-priority data prioritizes Fog nodes with higher remaining capacity, ensuring real-time processing without the need for task offloading or delays. The choice of remaining computing capacity as the main factor enables medium-priority data to make fast decisions for real-time processing. On the other hand, low-priority data are assigned to Fog nodes with lower remaining computing capacity since their real-time processing requirements are less critical compared to medium-priority data.

When the remaining energy or computing capacity of a selected Fog node falls below a threshold value of 25%, the data are rerouted from one Fog node to another or to the Cloud. The computation of a Fog node’s remaining computing capacity is determined by Equation (3).

## 5. Experimental Setup

Eclipse and MATLAB are used to validate MFHS.

MATLAB generates the fuzzy outputs to determine the data priority and the resource allocation based on fuzzy rules. 

Eclipse simulates the process environment (Edge, Mist, Fog, and Cloud) and calculates the power consumption, allocation time, and processing time. 

**Figure 13:**
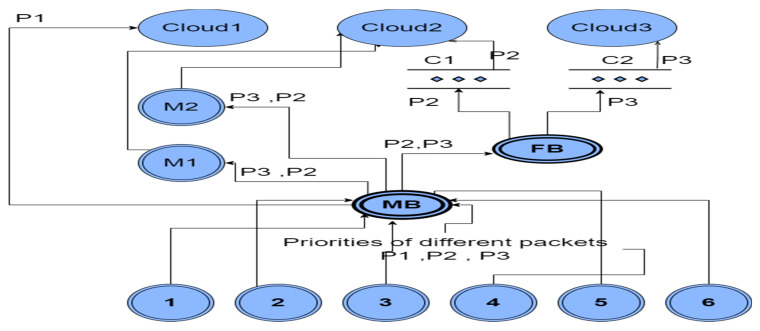
Work sequence.

In the experiment, six sensors are used at the Edge layer: two for the body temperature, two for the heart rate, and two for the glucose level. Two Mist nodes and one Mist broker are used at the Mist layer. Six Fog nodes and one Fog broker are used at the Fog layer, and three central Clouds are utilized at the Cloud layer. Table 4 summarizes the tools employed in the experiments and Figure 13 shows the process sequencing, with the node notation specified in Table 5.

In Figure 13, six Fog nodes are formed into two clusters based on the computational capacity (CC). Each cluster consists of several Fog nodes. Cluster one (C1) represents Fog nodes with a higher CC, and cluster two (C2) represents Fog nodes with a lower CC. 

For an illustration, let us assume in Table 6 that we have six Fog nodes, namely F1, F2, F3, F4, F5, and F6, with their respective CC. Based on our clustering principle, C1 will consist of F1, F4, and F6, while C2 will contain F2, F3, and F5. Accordingly, C1 is responsible for receiving the medium-priority data from the Fog broker, and C2 is responsible for receiving the low-priority data from the Fog broker.

The Fog clusters continue to process data until the CC of any Fog node goes below a threshold value of 25% of its capacity; when that happens, the data will be offloaded to the Cloud to avoid damaging the node.

## 6. Evaluation 

Our experimentation involves two metrics: the total energy consumption and the processing time. 

The total energy consumption (Etotal) consists of three different types of energy: the transmission of the packets (Etr); the classification of the packets (Ec) into high, medium, and low priority; and the resource allocation of the categorized packets (Ea) as follows:

(4) Etotal=Etr+Ec+Ea

Etr is calculated based on the size of the packets as in Equation (5):

(5) $$Etr=\sum_{i=1}^{n}Sn\times Eob$$

where Eob is the energy cost of transmitting one byte for a single hop, n is the total of transmitted packets, and Sn is the size of the n packet. In this experiment, the energy cost of transmitting one byte of packets for a single hop is assumed to be 0.5 mJ.

In a similar manner, the total processing time (Ttotal) is determined by considering the same factors as the total energy cost. It can be calculated using the following equation:

(6) Ttotal=Ttr+Tc+Ta

where Ttr represents the packet transmission time, Tc denotes the packet classification time, and Ta represents the packet allocation time.

## 7. Results

In our experimentation, we initially derive the total energy consumption and processing time metrics for our proposed MFHS model. Subsequently, we conduct a comparative analysis between the outcomes of MFHS and those of the feedback-based optimized fuzzy scheduling approach (FOFSA) algorithm, along with the adaptive task allocation technique (ATAT) and the osmosis load balancing (OLB) algorithm as documented in [15].

### 7.1. Energy Consumption

The energy consumption calculations are based on the addition of three parameters defined in Section 6: Etr, Ec, and Ea. 

#### 7.1.1. Etr Calculations

As a hardware implementation is absent in this context, the energy consumption calculations are derived through initialization with predefined values. This fixed value is determined by considering the energy consumption for transferring one bit of data at the data centre level, estimated to be approximately 0.2 mJ, as referenced in [24]. Consequently, the energy consumption for transferring a single byte of data amounts to 0.2 × 8 mJ, equalling 1.6 mJ.

The data transfer takes place across three tiers: Mist, Fog, and Cloud. Consequently, the total energy consumption for transmitting a byte of data aggregates to 1.6 × 3 mJ, which is equivalent to 4.8 mJ or approximately 0.0048 joule, and can be approximated as 0.005 joule. With the data packet size fixed at 1500 bytes, a value that optimizes TCP connection performance as outlined in [25], the energy consumption (Et) required to transmit a 1500-byte packet amounts to 0.005 × 1500 joule, which translates to 7.5 joule.

Extending this computation to encompass multiple packet transfers, the energy consumption for transmitting 20 packets (Et for 20 packet transfer) is determined as 20 × 7.5 joule, which equals 150 joule or approximately 0.15 KJ. Analogously, for 40, 60, 80, and 100 packet transfers, the corresponding energy consumptions (ET) amount to 300 joule (0.3 KJ), 450 joule (0.45 KJ), 600 joule (0.60 KJ), and 750 joule (0.75 KJ), respectively.

#### 7.1.2. Ec and Ea Calculations

Upon executing the MATLAB code for health condition assessment, employed for determining data priority through fuzzy-based classification, we noted an execution time of 0.35 s. Likewise, the execution of the code for fuzzy-based server allocation yielded an execution time of 0.34 s. Consequently, the cumulative processing time for both classification and allocation processes tallies to 0.35 + 0.34 = 0.69 s.

The power consumption of the CPU hinges on the configuration of a laptop equipped with an 11th Gen Intel(R) Core(TM) i7-1165G7 @ 2.80 GHz 1.69 GHz, with a power consumption spectrum spanning 12 to 28 watts. Considering the peak power consumption of 28 watts, equivalent to 100.8 KJ per hour, the energy consumption for running the code within 0.69 s is computed as (100.8 × 0.69)/3600 KJ, amounting to 0.019 KJ. This signifies the energy consumed for a single packet categorization, encompassing the determination of priority and its allocation to a specified node.

Extending this computation to accommodate multiple packets, the energy consumption for 20 packets totals to 0.019 × 20 KJ, equivalent to 0.38 KJ. Similarly, for 40 packets, it stands at 0.76 KJ, for 60 packets at 1.14 KJ, for 80 packets at 1.52 KJ, and for 100 packets at 1.9 KJ.

In summary, the overall energy consumption (Etotal) of the MFHS is tabulated as follows: 0.53 KJ for 20 packets, 1.06 KJ for 40 packets, 1.59 KJ for 60 packets, 2.12 KJ for 80 packets, and 2.65 KJ for 100 packets, as presented in Table 7.

Based on the results in [15], the energy consumption of FOFSA, ATAT, and OLB using the same packets transfer 20, 40, 60, 80, and 100 are shown in Table 8—please refer to Appendix A for detailed calculations.

#### 7.1.3. Energy Cost Comparison

**Figure 14:**
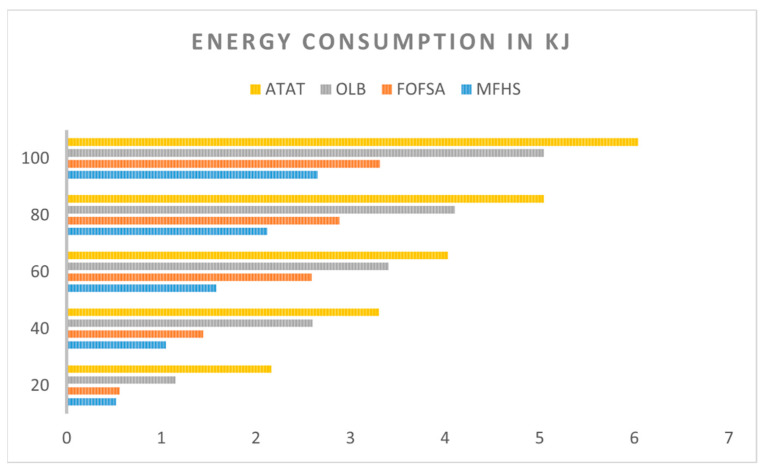
A comparative analysis of energy consumption.

Figure 14 presents a comparison of the energy costs between our proposed MFHS approach and the FOFSA, OLB, and ATAT algorithms discussed in [15], with respect to the number of packets. The chart clearly illustrates that our MFHS approach achieves superior energy cost results compared to the existing methods, particularly as the number of packets increases. This indicates that the MFHS algorithm outperforms its counterparts in handling extensive data analysis, which is essential for IoT networks.

### 7.2. Processing Time

The processing time computations are based on the three parameters defined in Section 6: Ttr, Tc, and Ta.

#### 7.2.1. Ttr Calculations

In our experimentation with the proposed MFHS using MATLAB, the total energy consumption for transferring one byte of data equates to 1.6 × 3 mJ, resulting in 4.8 mJ or approximately 0.0048 joule, which can be approximated as 0.005 joule. In parallel, considering a maximum CPU power consumption of 28 watts, translating to 100.8 KJ per hour, and with a packet size of 1500 bytes, we deduce that the average energy required for one byte packet transfer is approximately 0.0049 joule or 0.0049/1000 KJ.

Furthermore, it is evident that 100.8 KJ of energy is consumed within 1 h, corresponding to 3600 s. Therefore, 1 KJ of energy is consumed in 3600/100.8 s. Consequently, the energy consumption of 0.0049/1000 KJ amounts to (3600 × 0.0049)/(100.8 × 1000) seconds, multiplied by the packet size of 1500 bytes, which results in approximately 0.2625 s.

#### 7.2.2. Tc and Ta Calculations

The classification process takes 0.35 s, while the allocation process consumes 0.34 s, as elucidated in the above energy consumption calculation section. 

**Figure 15:**
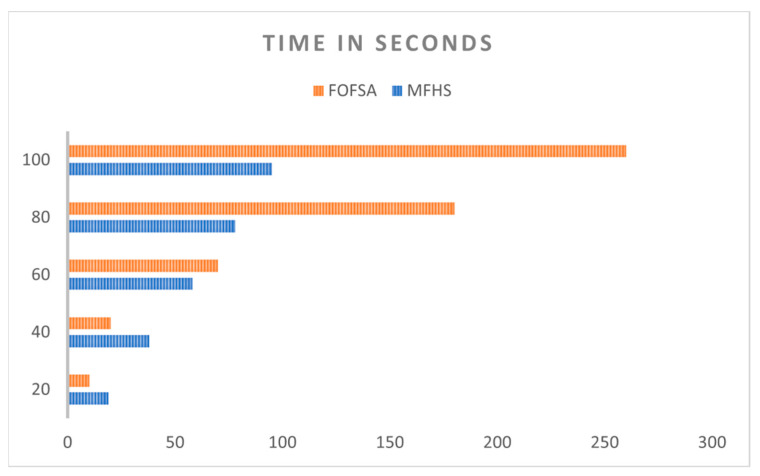
A comparative analysis of processing time.

In Figure 15, we present a comparison of the processing time between our proposed MFHS algorithm and the FOFSA method, which has been demonstrated to outperform both ATAT and OLB algorithms [15], with respect to the number of packets. The chart clearly indicates that the processing time of the MFHS algorithm outperforms the existing approach, particularly as the number of packets increases. This suggests that the MFHS algorithm excels in handling big data analyses, which are crucial for IoT networks. This comparison is made without sacrificing generality, highlighting the superior performance of MFHS in terms of processing time.

## 8. Discussion

Central to the crux of this study is a novel approach meticulously crafted to address the pivotal facets of offloading and load balancing, recognizing their cardinal significance in fortifying the performance metrics of diverse computing networks. The paramount goal of this innovative approach is to harness the latent potential of these key elements, thereby ushering in an era of heightened operational efficiency and enhanced system performance.

The strength of this approach lies in the adept utilization of fuzzy logic systems, acting as a guide in the areas of data processing, offloading strategies, and the intricate balance of workload distribution within the IoT networks. By harnessing the nuanced capabilities of fuzzy logic, the proposed framework brings forth a level of granularity and adaptability in the IoT landscape.

The proposed MFHS (Mist-based fuzzy healthcare system) systems unfold their operations at the very fabric of the Mist layer. This strategic initiation sets the tone for proactive decision making, injecting an element of timeliness and precision throughout the entire network. Here, the focal aim is a dual-pronged enhancement: the augmentation of energy efficiency and the amplification of processing expediency. By intervening at the nascent stages, MFHS sets the stage for a cascading series of optimized decisions that cumulatively foster an environment of superior performance.

The empirical validation of this paradigm-shifting approach is revealed through a meticulous evaluation conducted on the robust platforms of Eclipse and MATLAB. The results of this comprehensive assessment unequivocally showcase the tangible benefits reaped from the implementation of MFHS. Key performance indicators, namely processing time and power consumption, experience marked reductions, reinforcing the pivotal role played by this approach in fostering efficiency gains.

A noteworthy aspect is the empirical comparison against established benchmarks. Notably, the MFHS approach emerges triumphant, demonstrating its prowess over contemporaneous algorithms such as the feedback-based optimized fuzzy scheduling approach (FOFSA), the adaptive task allocation technique (ATAT), and the osmosis load balancing algorithm (OLB). This superiority manifests resoundingly across the twin dimensions of energy efficiency and processing time, affirming the innovative approach’s strength and its potential to revolutionize the IoT landscape.

## 9. Conclusions

The proposed approach in this study focuses on the key aspects of offloading and load balancing, recognizing their potential to enhance the performance of any computing network. To achieve this, fuzzy logic systems were employed to aid in processing, offloading, and workload balancing within IoT networks. The proposed MFHS systems initiate operations at the Mist level, enabling early decision making and aiming to improve both energy efficiency and processing time. The evaluation of MFHS involved the use of Eclipse and MATLAB, and the results demonstrated successful reductions in processing time and power consumption. Notably, the MFHS approach outperformed the feedback-based optimized fuzzy scheduling approach (FOFSA) algorithm, as well as the adaptive task allocation technique (ATAT) and osmosis load balancing algorithm (OLB), in terms of energy efficiency and processing time.

In conclusion, the proliferation of the Internet of Things (IoT) has ushered in a new era of connectivity and innovation, fostering seamless communication between devices and systems across diverse sectors. In healthcare, IoT networks hold the promise to reshape patient care, expedite critical interventions, and streamline administrative operations. As the demand for IoT networks continues to surge, the introduction of novel solutions like the Mist-based fuzzy healthcare system (MFHS) underscores the commitment to enhancing the efficiency, stability, and adaptability of IoT technology. By strategically allocating IoT data resources through the Mist layer, MFHS contributes to faster processing times, increased energy efficiency, and overall improved performance within IoT networks, thereby advancing the capabilities of healthcare systems and various other industries alike.

## Figures and Tables

**Table 1:** The reading of the medical sensors.

BT (Body Temperature)	HR (Heart Rate)	GL (Glucose Level)
Low 35.5 °C to 36.5 °C	Slow (<70 bpm)	Less (<60 mg/dL)
Normal 36.1 °C to 37.2 °C	Average 60 bpm to 110 bpm	Normal 50 mg/dL to 140 mg/dL
High 37 °C to 38 °C	Fast 100 bpm to 140 bpm	High 130 mg/dL to 240 mg/dL

**Table 2:** The fuzzy rules for data categorization.

BT (Body Temperature)	HR (Heart Rate)	GL (Glucose Level)	Health Score	Patient Health Condition	Data Priority
Low	Slow	Less	Poor	Critical	High
Low	Slow	Normal	Medium	Exposed to diseases	Medium
Low	Slow	High	Poor	Critical	High
Low	Average	Less	Medium	Exposed to diseases	Medium
Low	Average	Normal	Good	Healthy	Low
Low	Average	High	Medium	Exposed to diseases	Medium
Low	Fast	Less	Poor	Critical	High
Low	Fast	Normal	Medium	Exposed to diseases	Medium
Low	Fast	High	Poor	Critical	High
Normal	Slow	Less	Medium	Exposed to diseases	Medium
Normal	Slow	Normal	Good	Healthy	Low
Normal	Slow	High	Medium	Exposed to diseases	Medium
Normal	Average	Less	Good	Healthy	Low
Normal	Average	Normal	Good	Healthy	Low
Normal	Average	High	Good	Healthy	Low
Normal	Fast	Less	Medium	Exposed to diseases	Medium
Normal	Fast	Normal	Good	Healthy	Low
Normal	Fast	High	Medium	Exposed to diseases	Medium
High	Slow	Less	Poor	Critical	High
High	Slow	Normal	Medium	Exposed to diseases	Medium
High	Slow	High	Poor	Critical	High
High	Average	Less	Medium	Exposed to diseases	Medium
High	Average	Normal	Good	Healthy	Low
High	Average	High	Medium	Exposed to diseases	Medium
High	Fast	Less	Poor	Critical	High
High	Fast	Normal	Medium	Exposed to diseases	Medium
High	Fast	High	Poor	Critical	High

**Table 3:** The fuzzy rules for server allocation.

Data Priority	Computational Capacity of Mist Node	Source Allocation
High	High	Cloud
High	Medium	Cloud
High	Low	Cloud
Medium	High	Mist
Medium	Medium	Mist
Medium	Low	Fog
Low	High	Mist
Low	Medium	Fog
Low	Low	Fog

**Table 4:** System configuration.

Parameters	Configuration
Processor	11th Gen Intel(R) Core(TM) i7-1165G7 @ 2.80 GHz 1.69 GHz
Language	Java
Integrated Development Environment (IDE)	Eclipse
Development Kit	Java Development Kit (JDK) 17
Fuzzy rules integration	MATLAB

**Table 5:** Nodes’ description.

Nodes	Description
1, 2, 3, 4, 5, 6	BT, HR, GL nodes at the Edge layer
MB	Mist broker
FB	Fog broker
F1, F2, F3, F4, F5, F6	Nodes at the Fog layer
CL1, CL2, CL3	Nodes at the Cloud layer
C1 and C2	The two Fog clusters
M1 and M2	Mist nodes
CC	Computational capacity

**Table 6:** Fog nodes’ computational capacity.

Node Name	Computational Capacity
F1	360
F2	60
F3	120
F4	280
F5	40
F6	160

**Table 7:** Energy consumption of the MFHS for 20, 40, 60, 80, and 100 packets.

Number of Packets	E_tr_	E_c_ + E_a_	Etotal
20	0.15 KJ	0.38 KJ	0.53 KJ
40	0.3 KJ	0.76 KJ	1.06 KJ
60	0.45 KJ	1.14 KJ	1.59 KJ
80	0.60 KJ	1.52 KJ	2.12 KJ
100	0.75 KJ	1.9 KJ	2.65 KJ

**Table 8:** Total energy consumption (Etotal) of FOFSA, ATAT, and OLB.

Number of Packets	OLB	FOFSA	ATAT
20	1.15 KJ	0.56 KJ	2.16 KJ
40	2.6 KJ	1.44 KJ	3.3 KJ
60	3.4 KJ	2.5 KJ	4.03 KJ
80	4.1 KJ	2.8 KJ	5 KJ
100	5 KJ	3.3 KJ	6 KJ

## Data Availability

Not applicable.

## Appendix A

### Appendix A.1. Energy Consumption Calculations for FOFSA, ATAT, and OLB

#### Appendix A.1.1. FOFSA

The energy consumption of FOFSA [15] translates to approximately 4 KWH for 50 VMs, 10 KWH for 100 VMs, 18 KWH for 150 VMs, 20 KWH for 200 VMs, 23 KWH for 250 VMs, and 28 KWH for 300 VMs.

Our analysis of the execution time graph for VMs handling different numbers of tasks, as presented in [15], indicates that a single VM can execute a maximum of 10,000 tasks. Therefore, a VM can handle up to 10,000 tasks.

Consequently, the total number of tasks allotted equates to 500,000 for 50 VMs, 1,000,000 for 100 VMs, 1,500,000 for 150 VMs, 2,000,000 for 200 VMs, and 2,500,000 for 250 VMs.

Based on this analysis, we deduce the following energy consumption figures: 4 KWH for 500,000 tasks, 10 KWH for 1,000,000 tasks, 18 KWH for 1,500,000 tasks, 20 KWH for 2,000,000 tasks, and 23 KWH for 2,500,000 tasks.

Since the number of tasks has been considered equivalent to the number of packets, the energy consumption for each task aligns with the corresponding packet count. Thus, for 500,000 packets, the energy consumption amounts to 4 KWH, which translates to (4 × 20)/500,000 KWH, or (4 × 3600 × 20)/500,000 KJ, equalling 0.56 KJ.

Similarly, for 1,000,000 packets, the energy consumption corresponds to 10 KWH, translating to (10 × 40)/1,000,000 KWH, or (10 × 40 × 3600)/1,000,000 KJ, equalling 1.44 KJ.

Continuing this trend, for 1,500,000 packets, the energy consumption amounts to 18 KWH, which translates to (18 × 60)/1,500,000 KWH, or (18 × 60 × 3600)/1,500,000 KJ, resulting in 2.592 KJ.

For 2,000,000 packets, the energy consumption corresponds to 20 KWH, translating to (20 × 80)/2,000,000 KWH, or (20 × 80 × 3600)/2,000,000 KJ, resulting in 2.88 KJ.

Lastly, for 2,500,000 packets, the energy consumption amounts to 23 KWH, which translates to (23 × 100)/2,500,000 KWH, or (23 × 100 × 3600)/2,500,000 KJ, resulting in 3.312 KJ.

#### Appendix A.1.2. ATAT

Since the number of tasks has been equated to the number of packets, the energy consumption outcome for tasks in ATAT [15] will equivalently apply to the corresponding number of packets. Thus, for 500,000 packets, the energy consumption corresponds to 15 KWH. For instance, for a mere 20 packets, the energy consumption is calculated as (15 × 20)/500,000 KWH, which converts to (15 × 3600 × 20)/500,000 KJ, resulting in 2.16 KJ.

Similarly, with 1,000,000 packets, the energy consumption aligns with 23 KWH. For 40 packets, the energy consumption computes as (23 × 40)/1,000,000 KWH or (23 × 40 × 3600)/1,000,000 KJ, amounting to 3.3 KJ.

Furthermore, for 1,500,000 packets, the energy consumption value corresponds to 28 KWH. Consequently, for 60 packets, the energy consumption is determined as (28 × 60)/1,500,000 KWH or (28 × 60 × 3600)/1,500,000 KJ, resulting in 4.03 KJ.

Likewise, with 2,000,000 packets, the energy consumption value aligns with 35 KWH. For 80 packets, the energy consumption computation stands at (35 × 80)/2,000,000 KWH or (35 × 80 × 3600)/2,000,000 KJ, leading to 5.04 KJ.

Similarly, for 2,500,000 packets, the energy consumption corresponds to 42 KWH. Correspondingly, for 100 packets, the energy consumption calculates as (42 × 100)/2,500,000 KWH or (42 × 100 × 3600)/2,500,000 KJ, resulting in 6.04 KJ.

This pattern continues, extending the computation for larger numbers of packets, which demonstrates a consistent relationship between energy consumption and the number of tasks, underscoring the scalability of the presented results.

#### Appendix A.1.3. OLB

Given the equivalence between the number of tasks and the number of packets, the energy consumption results for tasks in OLB [15] naturally apply to their corresponding number of packets. Thus, for 500,000 packets, the energy consumption is reflective of 8 KWH. For example, when considering a mere 20 packets, this energy consumption translates to (8 × 20)/500,000 KWH, which further converts to (8 × 3600 × 20)/500,000 KJ, resulting in 1.15 KJ.

Similarly, for 1,000,000 packets, the energy consumption aligns with 18 KWH. Thus, for 40 packets, the energy consumption calculation stands at (18 × 40)/1,000,000 KWH or (18 × 40 × 3600)/1,000,000 KJ, yielding 2.6 KJ.

In a similar vein, when dealing with 1,500,000 packets, the energy consumption approximates 24 KWH. Consequently, for 60 packets, the energy consumption computes as (24 × 60)/1,500,000 KWH, which equates to (24 × 60 × 3600)/1,500,000 KJ, culminating in 3.4 KJ.

Similarly, for 2,000,000 packets, the energy consumption aligns with 29 KWH. Correspondingly, for 80 packets, the energy consumption computation stands at (29 × 80)/2,000,000 KWH or (29 × 80 × 3600)/2,000,000 KJ, yielding 4.1 KJ.

Finally, for 2,500,000 packets, the energy consumption is in the vicinity of 35 KWH. Correspondingly, for 100 packets, the energy consumption translates to (35 × 100)/2,500,000 KWH, resulting in (35 × 100 × 3600)/2,500,000 KJ, amounting to 5.04 KJ.

This coherent pattern persists, demonstrating a consistent relationship between energy consumption and the number of tasks (or packets), highlighting the scalability and predictability of the presented results.
